# Analysis of healthcare data security with DWT-HD-SVD based-algorithm invisible watermarking against multi-size watermarks

**DOI:** 10.1038/s41598-024-61479-4

**Published:** 2024-05-10

**Authors:** Himanshi Chaudhary, Virendra P. Vishwakarma

**Affiliations:** 1 University School of Information, Communication and Technology, Guru Gobind Singh Indraprastha University, Sector 16-C, Dwarka, New Delhi, India; 2 Department of Computer Science and Engineering, KIET Group of Institutions, Delhi-NCR, Ghaziabad, India

**Keywords:** Health care, Medical research, Engineering

## Abstract

In the modern day, multimedia and digital resources play a crucial role in demystifying complex topics and improving communication. Additionally, images, videos, and documents speed data administration, fostering both individual and organizational efficiency. Healthcare providers use tools like X-rays, MRIs, and CT scans to improve diagnostic and therapeutic capacities, highlighting the importance of these tools in contemporary communication, data processing, and healthcare. Protecting medical data becomes essential for maintaining patient confidentiality and service dependability in a time when digital assets are crucial to the healthcare industry. In order to overcome this issue, this study analyses the DWT-HD-SVD algorithm-based invisible watermarking in medical data. The main goal is to verify medical data by looking at a DWT-based hybrid technique used on X-ray images with various watermark sizes (256*256, 128*128, 64*64). The algorithm’s imperceptibility and robustness are examined using metrics like Peak Signal-to-Noise Ratio (PSNR) and Structural Similarity Index (SSIM) and are analyzed using Normalized Connection (NC), Bit Error Rate (BER), and Bit Error Rate (BCR) in order to evaluate its resistance to various attacks. The results show that the method works better with smaller watermark sizes than it does with larger ones.

## Introduction

The swift growth of digital media and the ease of information sharing have made data integrity, copyright protection, and authenticity issues crucial. The digital technology^1^ has brought unparalleled access and distribution, but it also risks misuse, unauthorized reproduction, and data manipulation. As invention and duplication blend, effective intellectual property protection and digital transaction trust solutions are more important than ever. Photographs can reveal personal information, while designs and schematics can reveal company data. Image security^2^ breaches can lead to identity theft, corporate espionage, and other privacy and commercial breaches. To prevent such risks, photos must be secure. Digital manipulation has made it harder to tell modified photographs from real ones. Digital watermarks and cryptographic signatures can provide an unbreakable link between the image and its source, boosting confidence in the image’s authenticity and source. In medicine, photographs are crucial to diagnosis, treatment, and research, making image security crucial^3,4^. X-rays, MRIs, and CT scans provide vital diagnostic information for healthcare decisions. These photos must be secure to ensure patient privacy and medical data accuracy. Medical images reveal the body’s internal structures and problems. These photos are crucial to accurate diagnosis. Medical image tampering^5^ can cause misdiagnosis, incorrect treatment, and patient harm. Healthcare practitioners can make educated decisions that affect patient outcomes by protecting medical images. Personal identities and medical histories are sensitive in medical imaging. Keeping these photographs secure is lawful, ethical, and essential for patient trust. Strong security measures safeguard patient privacy, medical information, and unauthorized access.

Digital watermarks^6–8^—an amazing combination of technology and information security—are a powerful weapon for the digital era. Invisible Digital watermarks^9–11^ are invisible patterns or codes that are effortlessly inserted into photos, sounds, movies, and documents. The concealed identification typically reveals the content’s origin, ownership, or usage rights. These watermarks are invisible to the human eye but may be identified and extracted using specialised software to verify digital content’s validity and ownership. From preventing cash counterfeiting to protecting digital creative works, digital watermarking has had a fascinating history. This technology is driven by computer science, cryptography, and signal processing advances. Each step has made digital watermarks more robust and versatile, adapting to different media types and solving a growing number of problems. This research study examines digital watermarks’ methods, applications, problems, and potential future.

By analysing this technology, we want to show how digital watermarks improve data security, copyright protection, and digital media. We want to help artists, researchers, and industries achieve safe and trustworthy digital interactions by deepening our understanding of its history, methods, and ramifications. Digital watermarks are essential for improving data integrity, patient privacy, and research credibility in the field of medical imaging, where accuracy is of the utmost importance. Digital watermarks protect medical images from manipulation and unauthorised changes by incorporating barely noticeable markers inside them. By securely inserting encrypted IDs, this technology not only prevents data intrusions but also protects patient confidentiality. Digital watermarks make traceability easier in longitudinal studies, upholding the validity of study findings. They also make it possible for healthcare professionals to collaborate and share protected images, which is essential for remote consultations and interdisciplinary conversations. Digital watermarks, which are compliant with regulatory requirements, serve as a crucial instrument to support confidence and accountability in medical imaging, ultimately supporting the basis of precise diagnoses and efficient treatments.

**Figure 1:**
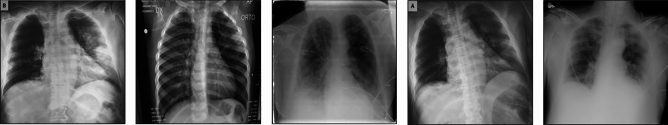
Input images.

This research focuses on a detailed quantitative assessment of important measures that evaluate the security of healthcare data. This paper includes five cover images, each of size 512 × 512 pixels, as illustrated in Fig. 1. 3 different sizes of watermark are used, as shown in Fig. 3. The Figs. 4, 5, 6, 7, 8 displays the entire quantitative analysis conducted on all cover photos with different watermark sizes. The methodology utilizes the DWT-HD-SVD strategy for all watermarking. This technique is specifically selected for its effectiveness in guaranteeing both resilience and invisibility in healthcare data applications. The succeeding sections elaborate on the intricate quantitative results, offering a detailed comprehension of the algorithm’s performance. In addition, to emphasize the algorithm’s effectiveness in various situations, the research includes a total of 12 different attacks. This comprehensive analysis aims to emphasize the algorithm’s robustness and imperceptibility in real-world situations when healthcare data security encounters any breach. This study provides a thorough quantitative analysis of both robustness and imperceptibility, utilizing the DWT-HD-SVD method. The results, along with a thorough analysis and examination of different attacks, enhance algorithm’s effectiveness in safeguarding healthcare data in current digital era.

## Related work

Invisible watermarking is crucial in today’s medical world^12^. Critical medical data, including patient records and photographs, are protected against tampering in order to maintain confidence. It protects patient privacy and improves data security by incorporating authentication and access information. This technology is essential for reliable medical imaging, the validity of clinical research, and the fight against fake medications. Additionally, it is essential for telemedicine^13^, HIPAA compliance, and monitoring data flow within healthcare organizations. Invisible watermarking continues to be a crucial tool for secure and reliable data management in the medical industry as healthcare relies more and more on digital solutions. Transformations^14,15^ are used to embed watermark depending on the type of media and the specific requirements of the application. Frequency domain and time domain approaches can be used to transform data^16^. Data can be hidden within frequency coefficients or sub-bands using frequency domain transformations, such as the Discrete Cosine Transform (DCT) and the Discrete Wavelet Transform (DWT)^17^, which are frequently used for image and audio watermarking. The watermark information is dispersed across the time domain of the signal using time domain transformations^18,19^, such as Spread Spectrum Techniques and the Discrete Fourier Transform (DFT) for audio. Spatial and temporal approaches are combined in video watermarking, and some techniques even adjust to the type of content. The application’s specific aims, whether copyright protection, content authentication, or data concealing, should be carefully considered when selecting the transformation method. These factors include watermark robustness, imperceptibility, and robustness. The selection of the best transformation and embedding techniques may be influenced by various media applications’ priorities on certain characteristics.^20^ presented two blind watermarking methods for safeguarding medical photos used in telemedicine. The techniques—which combine DWT and Schur decomposition or DCT and Schur decomposition—demonstrate robustness and imperceptibility, guaranteeing the highest quality of watermarked photos while preserving patient privacy and data confidentiality. Zeng et al.^21^ introduce a revolutionary zero watermarking methodology to improve security. Medical image watermarks are embedded and extracted using KAZEDCT feature extraction, perceptual hashing, chaotic mapping encryption, and zero watermarking. Experimental results show the algorithm’s watermark extraction and resistance to common and geometric attacks, boosting medical image security. A block-based watermarking approach^22^ in the DWT-DCT domain uses the suggested PQIM technique. Based on PQIM, three AC components (AC(0, 1), AC(1, 0), and AC(1, 1)) are chosen for watermark embedding with picture quality-preserving settings. Robustness is improved by multiple-bit embedding with majority voting and modifying vector norms. While maintaining picture quality, experimental results reveal an advantage over 14 other approaches in DWT, SVD, or DCT domains. Future research will use variable subband use in the DWT architecture to make the schemes resistant to desynchronization attacks. This study^23^ uses a novel Optimal Amplitude Modulation (OAM) strategy to combine IWT with SVD to address the false positive problem (FPP) in SVD-based approaches. The OAM uses an Optimal Embedding Factor (OEF) and Target-Detection Optimization Mechanism (TDOM) to improve invisibility and resilience. The technique supports multiple watermark sizes and resists picture compression, noise addition, cropping, scaling, and sharpening.

Digital video watermarking and algorithm optimization will be studied. This scientific article^24^ presents a robust hybrid watermarking system for digital images that meets imperceptibility, robustness, security, and payload requirements. DCT, DWT, and SVD help the system attain these aims. Watermark encryption begins with Arnold map pixel position shuffling. DWT, SVD, and DCT are used on host and watermarked pictures. Create a watermarked image by embedding the watermark image. Extensive experiments test the system against various attacks. The results reveal that the suggested strategy is more resilient, undetectable, and secure than others. The authors stress the Arnold map’s watermark image security. This hybrid resilient watermarking method may safeguard digital images. The articles^25,26^ have utilised the medical image dataset for COVID-19 detection and other paper used machine learning on the EEG image dataset, these two papers can be further utilised as a trusted source of the dataset for watermarking purpose.

## Watermarking scheme

To conduct the analysis of the DWT-HD-SVD-based experiment, we utilized five input images^27^ shown in Fig. 1, all of which were grayscale lung images showing COVID infection. The experiment involved the different sizes of black and white watermarks, as depicted in Fig. 3. This watermark was employed in three sizes: 256x256, 128x128, and 64x64 pixels. Subsequently, all the input images were watermarked using each of these three watermark sizes. The analysis was carried out by assessing various parameters, including PSNR (Peak Signal-to-Noise Ratio), NC (Normalized Correlation), BER (Bit Error Rate), SSIM (Structural Similarity Index), and BCR (Bit Correct Rate). These parameters’ mathematical expressions and descriptions are provided in Table 1. These metrics were evaluated under two conditions: without any attack and with additional attacks, as described in Table 2. The subsequent sections present a detailed analysis.

**Table 1:** Description and mathematical expressions of metrics.

Metric name	Description mathematical	Expression
PSNR	Peak Signal-to-Noise Ratio, or SNR, compare the mean squared error (MSE) between an original image (I) and a watermarked image (K) to determine the highest pixel value that can be obtained (often 255 for an 8-bit image) in order to determine the quality of the image	$PSNR = 10 \cdot \log_{10} \left( {\frac{{255^{2} }}{{{\text{MSE}}}}} \right)$
NC	By calculating and normalizing the cross-correlation of two images (I and K), NC (Normalized Correlation) calculates how similar the two images are	$NC = \frac{{\sum \left( {I\left( x \right) \cdot K\left( x \right)} \right)}}{{\sqrt {\sum \left( {I\left( x \right)^{2} } \right) \cdot \sum \left( {K\left( x \right)^{2} } \right)} }}$
BER	The ratio of erroneous bits to the total number of bits in the image	$BER = \frac{{\text{Number of Bit Errors}}}{{\text{Total Bits in the Image}}}$
SSIM	Taking luminance, contrast, and structure into account, the SSIM (Structural Similarity Index) calculates the structural similarity between two images (I and K) *SSIM* = (2 *µI µK* + *C*1) (2*σIK* + *C*2) (*µI*2 + *µK*2 + *C*1)(*σI*2 + *σK*2 + *C*2) Where: *µI* and *µK* are the average pixel values of images I and K *σ*2 *I* and *σK*2 are the variances of images I and K *σIK* is the covariance between images I and K *C*1 and *C*2 are constants to stabilize the division	$SSIM = \frac{{\left( {2 \cdot {\upmu }_{I} \cdot {\upmu }_{K} + C_{1} } \right) \cdot \left( {2 \cdot {\upsigma }_{IK} + C_{2} } \right)}}{{\left( {{\upmu }_{I}^{2} + {\upmu }_{K}^{2} + C_{1} } \right) \cdot \left( {{\upsigma }_{I}^{2} + {\upsigma }_{K}^{2} + C_{2} } \right)}}$
BCR	The ratio of correctly transmitted bits to the total number of bits	$BCR = \frac{{\text{Number of Correctly Transmitted Bits}}}{{\text{Total Number of Bits in the Image}}}$

**Table 2:** Description and types of attacks.

Attack type	Description
No Attack	Watermarked image is not attacked in any manner
Average filter	The average filter attack smooths a watermarked image by averaging neighbourhood pixel values, which might damage its quality or make it less perceptible
Gaussian low-pass filters	Gaussian low-pass filters convolve images with Gaussian kernels. This method smooths the image and reduces noise and fine-grained characteristics. It is called “low-pass” because it passes low-frequency components like smooth gradients and huge structures while attenuating high-frequency components like sharp edges and noise
Gaussian noise	Pixel values vary randomly according to a Gaussian (normal) distribution in Gaussian noise. It causes random brightness and colour changes in images and degrades quality. Gaussian noise can affect a watermarked image in numerous ways, such as noise interference, reduced visibility, and quality degradation
Histogram equalization	Redistributing pixel intensity levels enhance image contrast and visibility with histogram equalization. A histogram equalization attack on a watermarked image may change its appearance and watermark visibility
JPEG compression	Applying JPEG compression to a watermarked image can result in data loss and can worsen the watermark’s quality and visibility. This is known as a JPEG compression assault
JPEG2000 compression	Applying JPEG2000 compression to a watermarked image constitutes a JPEG2000 compression attack. The data compression and modification techniques employed in JPEG2000 encoding may have an effect on the watermark’s quality and visibility
Median	A median attack on a watermarked image uses a median filter. The median (middle value) of pixel values in a neighbourhood is used by a median filter. This method reduces noise but can distort or eliminate the watermark depending on filter size and features
Motion blur	When watermarked images are intentionally or unintentionally moved during capture or post-processing, blurred patches develop. This happens when a camera or object is moving during a photo or when adding motion blur effects in image editing software. Motion blur can reduce watermark visibility in affected image areas
Salt and pepper noise	Salt and pepper attacks add random bright and dark pixels or noise to watermarked photos. Greyscale “salt” pixels have 255 intensity values, while “pepper” pixels have 0. The attack distorts images, making watermark identification and extraction difficult. Salt and pepper noise can blur details, degrade image quality, and disguise watermarks. Watermarking methods are resistant to image degradation, including salt and pepper noise, for accurate recognition and extraction
Sharpening attack	While a sharpening assault on a watermarked image brings out more details, it also increases visual artifacts and has the potential to alter the watermark
Speckle noise	Adding random, grainy pixels or noise to an image is known as a “speckle noise attack,” and it can be used to obfuscate watermarks and lower image quality

### Proposed methodology

**Figure 2:**
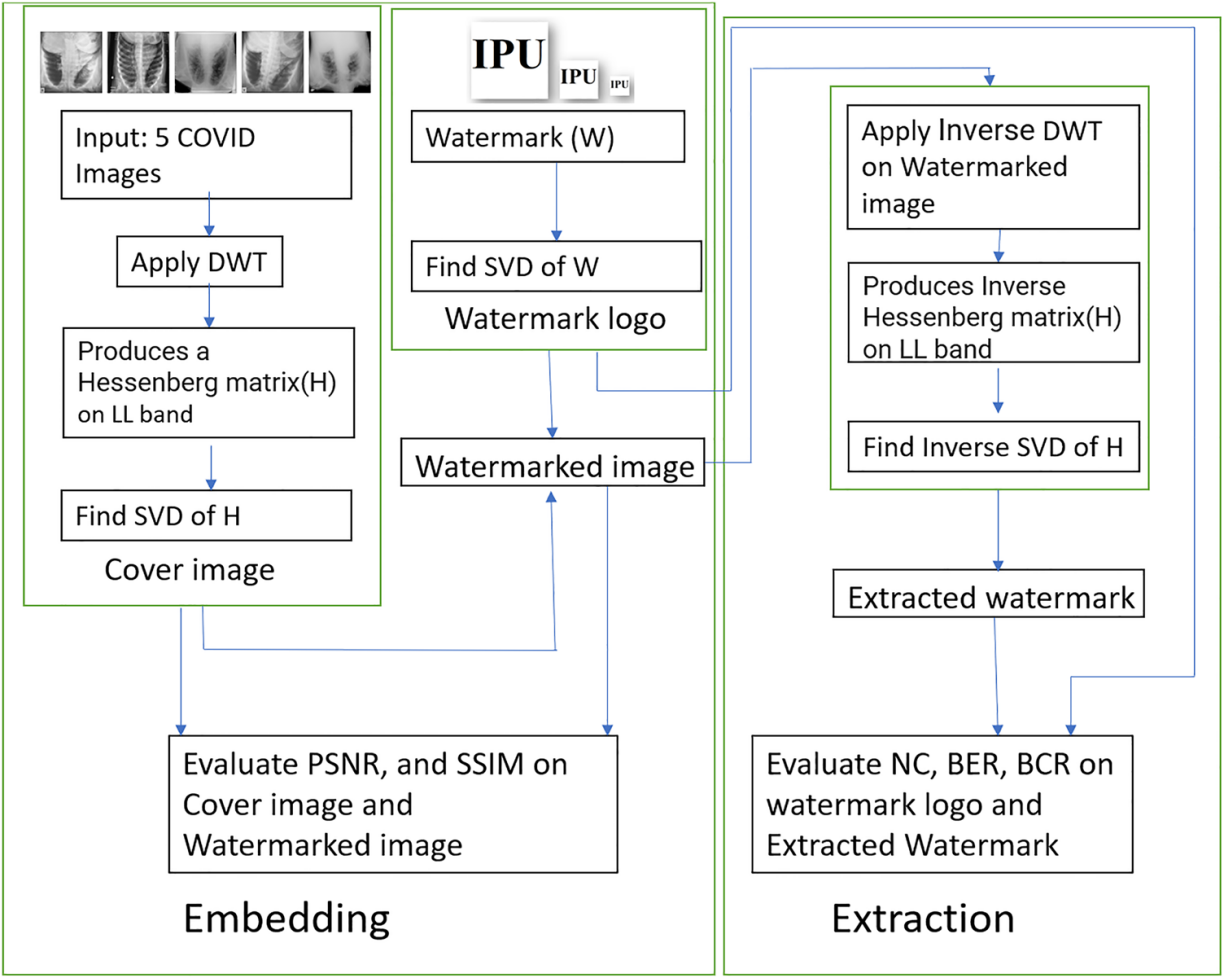
DWT-HD-SVD based algorithm description.

**Figure 3:**
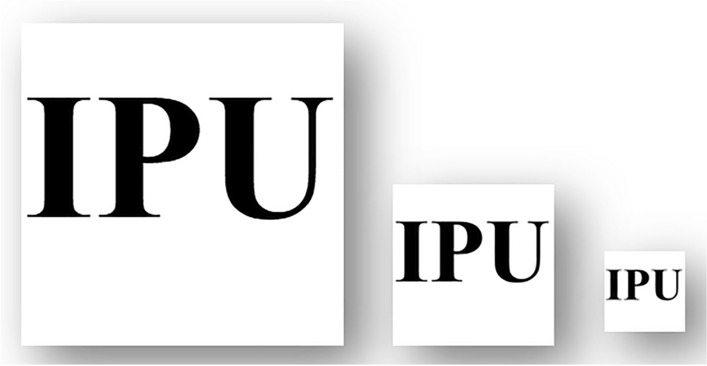
Watermark image in size (**a**) 256*256, (**b**) 128*128, (**c**) 64*64.

In this experiment, 5 input images are considered taken from data base^27^. All of these input images are applied algorithm DWT-HD-SVD^28^. The algorithm is shown in Fig. 2. This algorithm is implemented using Matlab R2021a and the results are produced using MS office 365. The images taken were 512*512 input images and each image is watermarked with three different size of black and white watermarks shown in Fig. 3. The algorithm used is explained Table 3 and pseudocode^29^ is defined in Table 4. DWT is used as a mathematical methodology employed to partition an image into numerous scales or levels. This process enables the extraction of pertinent insights into the image’s intricate features and structures across varying resolutions. This technique enables effective image compression, denoising, and analysis by capturing both high-frequency and low-frequency components of the image. The application of Hessenberg decomposition following the DWT on images results in the transformation of DWT coefficients into a structured format. This structured form facilitates efficient analysis and processing of images, particularly in the context of compression. The application of SVD on an image subsequent to DWT, followed by HD, and another SVD, presents a robust methodology for image compression and feature extraction. This technique effectively captures significant image patterns in a concise representation.

**Table 3:** Algorithm for embedding and attacking watermarks.

Algorithm steps
1. Load Cover Image and Watermark
2. Define method as ’DWT-HD-SVD’, Set alpha = 0.08
3. Define attacks
4. Loop over the list of attacks, including ’No Attack’
5. For every Cover image embed each watermark of size 256*256, 128*128, 64*64
6. For each attack scenario (including ’No Attack’), embed the watermark
7. Calculate NC, PSNR, SSIM, BER, and BCR, displaying results for each case

**Table 4:** Pseudocode.

Pseudocode
Step 1. Load or create images
Step 2. Display cover_image and watermark_logo
Step 3. Set method to ’DWT-HD-SVD’
Step 4. Set alpha = 0.08
Step 5. Define attacks = [’No Attack’, ’Gaussian low-pass filter’, …]
Step 6. Define params = [0, 3, 3, 0.001, …]
Step 7. For each attack in attacks Step 8. Get param for the current attack
Step 9. Apply watermarking, calculate NC, PSNR, SSIM, BER, and BCR
Step 10. End For

## Results and discussion

This section presents an analysis of five lung images affected by COVID-19, which are used as cover images shown in Fig. 1. These images are subjected to watermarking with three different sizes, as outlined in Fig. 3. The analysis aims to assess the watermarking process’s robustness by evaluating metrics like PSNR, SSIM. Additionally, imperceptibility is examined through metrics such as NC, BER, BCR. The watermarking algorithm employed is a hybrid approach based on DWT-HD-SVD, previously introduced in a research paper^28^. Subsequently, the extraction process is detailed, and the algorithm’s workflow is depicted in Fig. 2. To evaluate each metric, all attacks discussed in Table 2 are practised. The following section presents a comparative analysis of these performance metrics.

### Robustness and imperceptibility

The term “robustness” pertains to the capacity of an image watermarking or processing approach to endure diverse challenges or attacks while retaining the encoded information. Consider a scenario where a picture has been embedded with significant data through watermarking and subsequently undergoes compression, noise interference, or other modifications. A resilient watermarking system guarantees the preservation and recoverability of the watermark, even in the face of various obstacles. The act of fortifying one’s information with a robust shield serves to safeguard it against potential attacks. The importance of robustness cannot be overstated in applications such as data authentication and tamper detection. It is imperative to guarantee the integrity of embedded information, irrespective of external influences. In contrast, imperceptibility pertains to the preservation of the visual integrity and attractiveness of a picture. Consider the following scenario: you have applied a watermark or made certain modifications to an image, and you desire to ensure that these alterations are not overtly conspicuous to observers. The concept of imperceptibility is relevant in this context as it guarantees that these alterations are sufficiently subtle to evade detection by the human visual system. The act of effortlessly integrating a concealed message within a painting while preserving its inherent aesthetic appeal might be likened to an artistic endeavour. The attribute of imperceptibility holds significant importance in situations where the preservation of visual aesthetics and user experience is paramount, as observed in domains like art, photography, and medical imaging. The following metrics are frequently utilized:

1. **The Normalized Connection (NC) Metric**: The NC metric quantifies the degree of linear connection existing between the original image and the watermarked image. A rating in proximity to 1 signifies a significant level of resemblance and indistinguishably.
2. **The Structural Similarity Index (SSIM)**: The SSIM is a metric used to assess the degree of structural similarity between two images, specifically the original image and the watermarked image. SSIM considers various factors such as luminance, contrast, and structure in its evaluation. A rating in proximity to 1 indicates a heightened level of imperceptibility.
3. **The Peak Signal-to-Noise Ratio (PSNR)**: The PSNR is a metric used to quantify the relationship between the highest signal level and the noise level that arises because of the watermarking procedure. Greater PSNR readings are indicative of enhanced imperceptibility.
4. **The Bit Error Rate (BER)**: The BER is a metric used to measure the number of bits that have been communicated or modified wrongly in the watermarked image in comparison to the original image. Lower bit error rate (BER) values are indicative of increased imperceptibility.
5. **The Bit Change Rate (BCR)**: The BCR is a metric used to quantify the rate at which bits inside a watermarked image have been modified in relation to the original image. A reduced bit conversion rate (BCR) is indicative of enhanced imperceptibility.

In the following section, results are discussed.

### Comparison of Normalized Correlation

**Figure 4:**
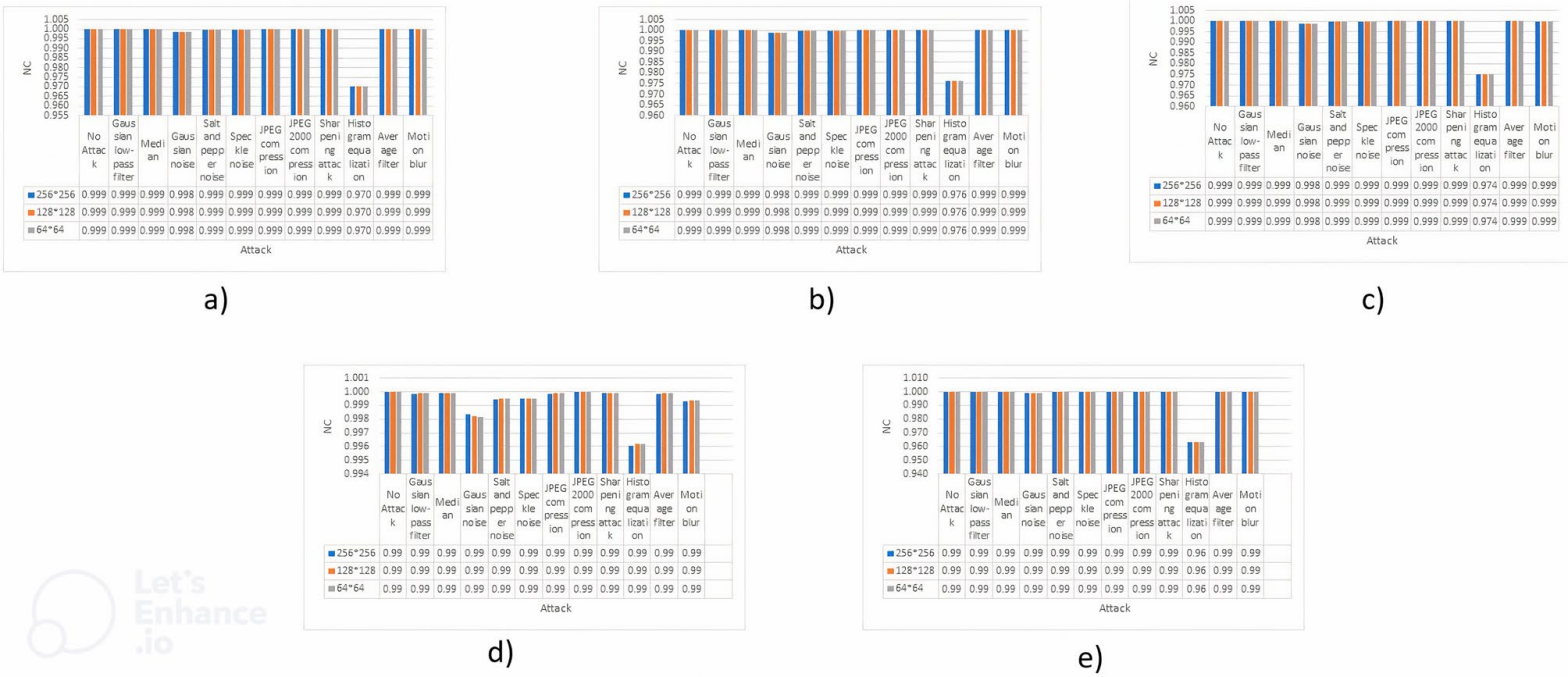
NC values are represented against each attack, values are also shown against all the mentioned attacks and no attack where (**a**) Image 1, (**b**) Image 2, (**c**) Image 3, (**d**) Image 4, (**e**) Image 5

In this section, the results of the Normalized Correlation (NC) evaluation will be presented. These evaluations were conducted on cover images shown in Fig. 1 that underwent watermarking using three different sizes of watermarks shown in Fig. 3. The NC metric plays a crucial role in assessing the imperceptibility and robustness of the watermark against several potential attacks. This paper provides a thorough analysis of the resilience of watermarked photos against various attacks mentioned in Table 2, while also examining their ability to preserve a strong association with the original content. The findings will be succinctly presented in Fig. 4, presenting the values as well as a graphical presentation.

The results shown in Fig. 4 clearly demonstrate the algorithm’s remarkable imperceptibility, as indicated by the graphs. In all the attack scenarios, the Normalized Correlation (NC) values continuously reach or close to the desired threshold of 1, indicating a significant level of imperceptibility. This level of imperceptibility is highly desirable in these circumstances. It is important to acknowledge, however, that there is an opportunity for enhancement, specifically in addressing the unfavourable outcomes discovered in the context of the Histogram Equalization attack. Results can be stated as follows: Results can be stated as follows:

- $$\bullet \,\forall {\text{Attacks}} \in \, \{ {\text{Table 3}}\} :{\text{ NC}} \approx {1}$$  
- $$\bullet \,\exists {\text{Attack }}:{\text{ Attack}} = {\text{Histogram equalization}}s.t.{\text{NC is not close to 1}}$$  

### Comparison of PSNR

**Figure 5:**
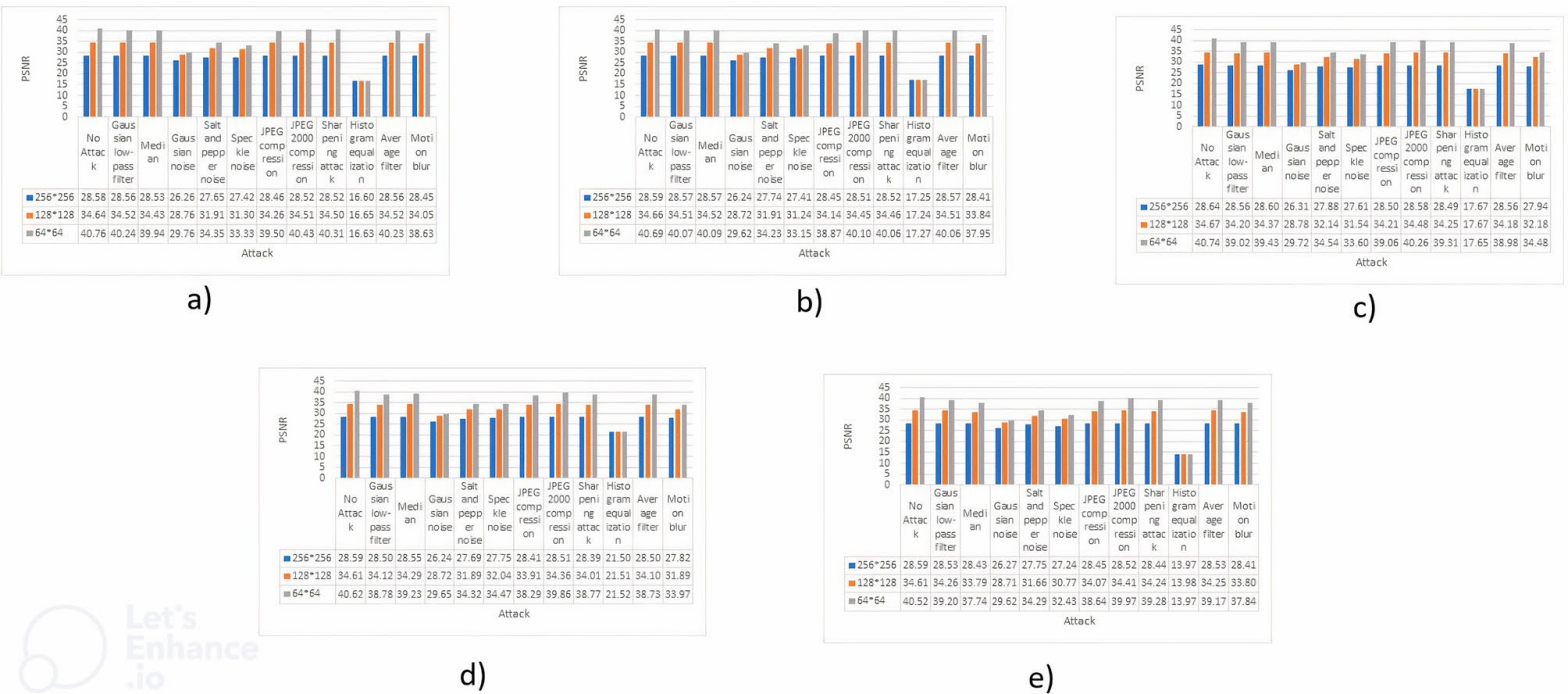
PSNR values are represented against each attack, values are also shown against all the mentioned attacks and no attack where (**a**) Image 1, (**b**) Image 2, (**c**) Image 3, (**d**) Image 4, (**e**) Image 5.

This section will explore the results obtained from Peak Signal-to-Noise Ratio (PSNR) assessments. The evaluations were conducted on the cover photos, as depicted in Fig. 1, after applying three different watermark sizes, as described in Table 2. The Peak Signal-to-Noise Ratio (PSNR) is a crucial statistic utilized in the field of image processing. It plays a significant role in evaluating the quality of watermarked images, specifically in terms of their accuracy to the original content. This study undertakes a thorough analysis of the resilience of watermarked photos against several types of attacks, as outlined in Table 1. Additionally, it evaluates their ability to maintain a high degree of similarity with the original source material. The results will be concisely displayed in Fig. 5, which will not only exhibit the numerical values but also offer a graphical depiction of the outcomes, providing a comprehensive perspective of the PSNR evaluations. Upon conducting an analysis of the outcomes obtained from the five input photos, it becomes apparent that there is a consistent upward trend in the PSNR (Peak Signal-to-Noise Ratio) value as the dimensions of the watermark decrease. This phenomenon remains consistent across different types of attacks, although it is important to note that histogram equalization stands out as a prominent exception. The general mathematical representation is as follows:

$$PSNR \propto 1/{\text{Size}}\_{\text{of}}\_{\text{Watermark}}$$

### Comparison of SSIM

**Figure 6:**
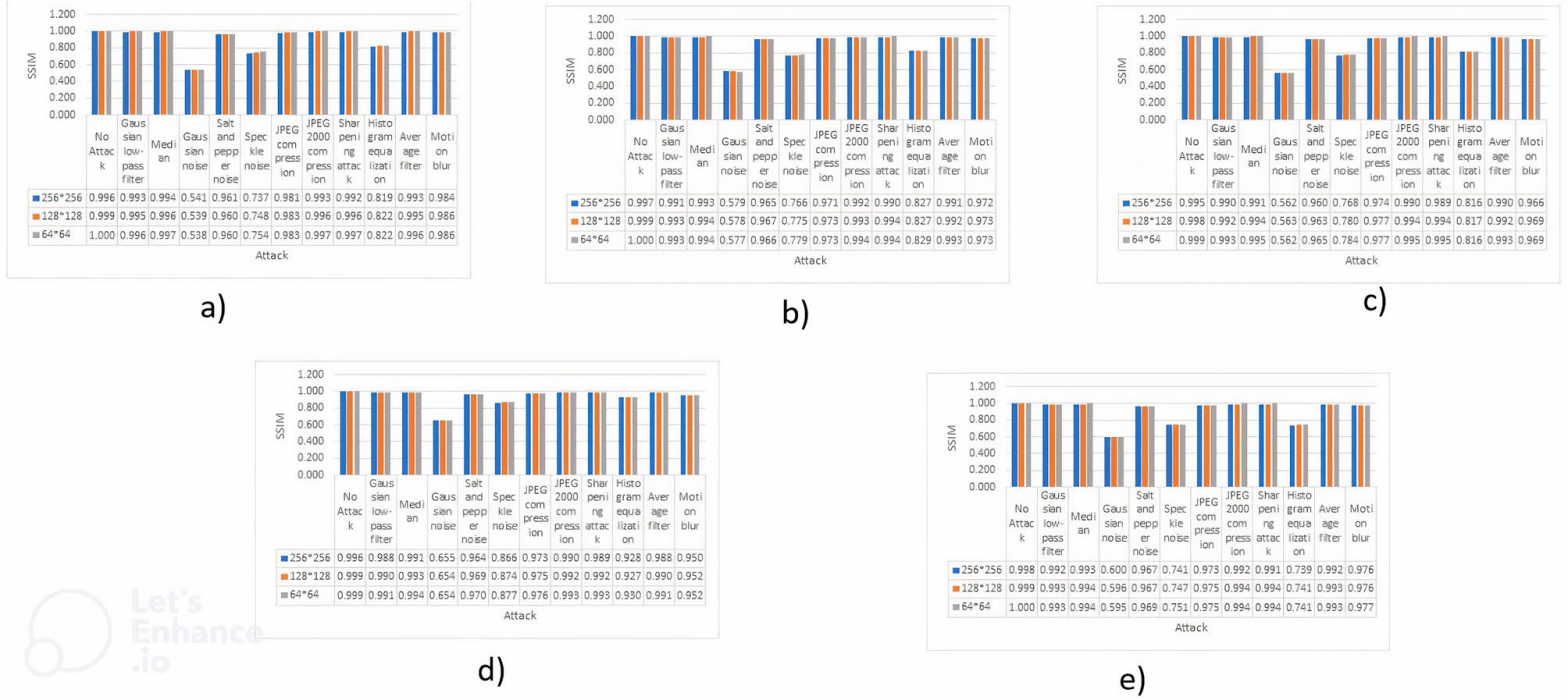
SSIM values are represented against each attack, values are also shown against all the mentioned attacks and no attack where (**a**) Image 1, (**b**) Image 2, (**c**) Image 3, (**d**) Image 4, (**e**) Image 5.

In this section, we shift our focus towards the examination of outcomes pertaining to the Structural Similarity Index (SSIM) study. The evaluations were conducted on the cover images, as shown in Fig. 1, after applying three different watermark sizes as described in Fig. 3. The Structural Similarity Index (SSIM), a fundamental metric for assessing picture quality, plays a significant role in measuring the similarity between watermarked images and their original content. This study conducts a thorough evaluation to determine the resilience of watermarked images against several potential attacks, as outlined in a comprehensive manner in Table 1. Furthermore, we examine their capacity to maintain a robust connection with the primary content. The results will be given in a Fig. 6, which will effectively encapsulate both numerical values and a graphical depiction of the Structural Similarity Index Measure (SSIM) evaluations. This will provide a full summary of the findings.

Upon conducting an analysis of the outcomes obtained from the five input photos, it becomes apparent that there is a consistent effect on SSIM value as the dimensions of the watermark decrease. This phenomenon remains consistent across different types of attacks, although it is important to note that Gaussian noise, Speckle noise, and histogram equalization stand out as exceptions. The general mathematical representation is as follows:

$$\forall {\text{Attacks}} \in {\text{Table 3 }}:{\text{ SSIM}} \approx {1}$$

$$\exists {\text{Attack }}:{\text{ Attack}} = {\text{Gaussian noise}},{\text{ Histogram equalization}},{\text{ and}}\;{\text{Speckle noise s}}.{\text{t}}.{\text{ SSIM is not close to 1}}$$

### Comparison of BCR

**Figure 7:**
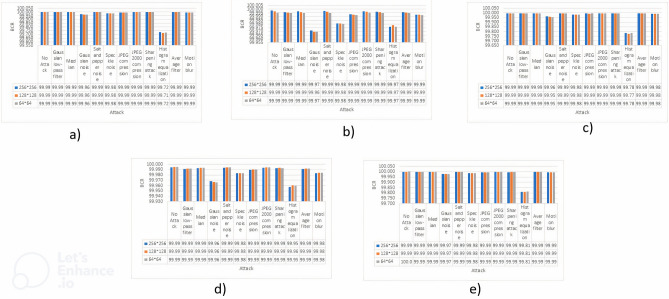
BCR values are represented against each attack, values are also shown against all the mentioned attacks and no attack where a(**a**) Image 1, (**b**) Image 2, (**c**) Image 3, (**d**) Image 4, (**e**) Image 5.

This section will now direct our attention towards the analysis of Bit Change Rate (BCR) findings. The evaluations were conducted on the cover photos, as depicted in Fig. 1, following the implementation of three different watermark sizes, as specified in Fig. 3. The Bit Correct Rate (BCR), a crucial parameter in the analysis of watermarking techniques, plays a significant role in assessing the degree to which watermarked images have been modified in relation to the original content. This study does a comprehensive assessment to measure the resilience of watermarked photos against different types of attacks, as outlined in Table 1, with careful precision. In addition, we investigate their ability to reduce modifications and maintain the authenticity of the original content. The findings of these evaluations will be concisely displayed in a Fig. 7, which will include both numerical values and a graphical representation of the BCR outcomes. This figure will offer a thorough depiction of the influence of the watermarking procedure on image alteration.

After conducting an examination of the results acquired from the five input images, it becomes seen that there is a consistent value close to 1 on BCR value. This effect becomes more pronounced as the dimensions of the watermark become smaller. This phenomenon is present across a wide variety of attacks; nonetheless, it is essential to point out that histogram equalization stands out as an exception to the rule.

The general mathematical representation can be written like this:

$$\begin{gathered} \forall {\text{Attacks}} \in \, \{ {\text{Table 3}}\} :{\text{ BCR}} \approx {1} \hfill \\ \exists {\text{Attack }}:{\text{ Attack}} = {\text{Histogram equalization s}}.{\text{t}}.{\text{ BCR is not close to 1}} \hfill \\ \end{gathered}$$

### Comparison of BER

**Figure 8:**
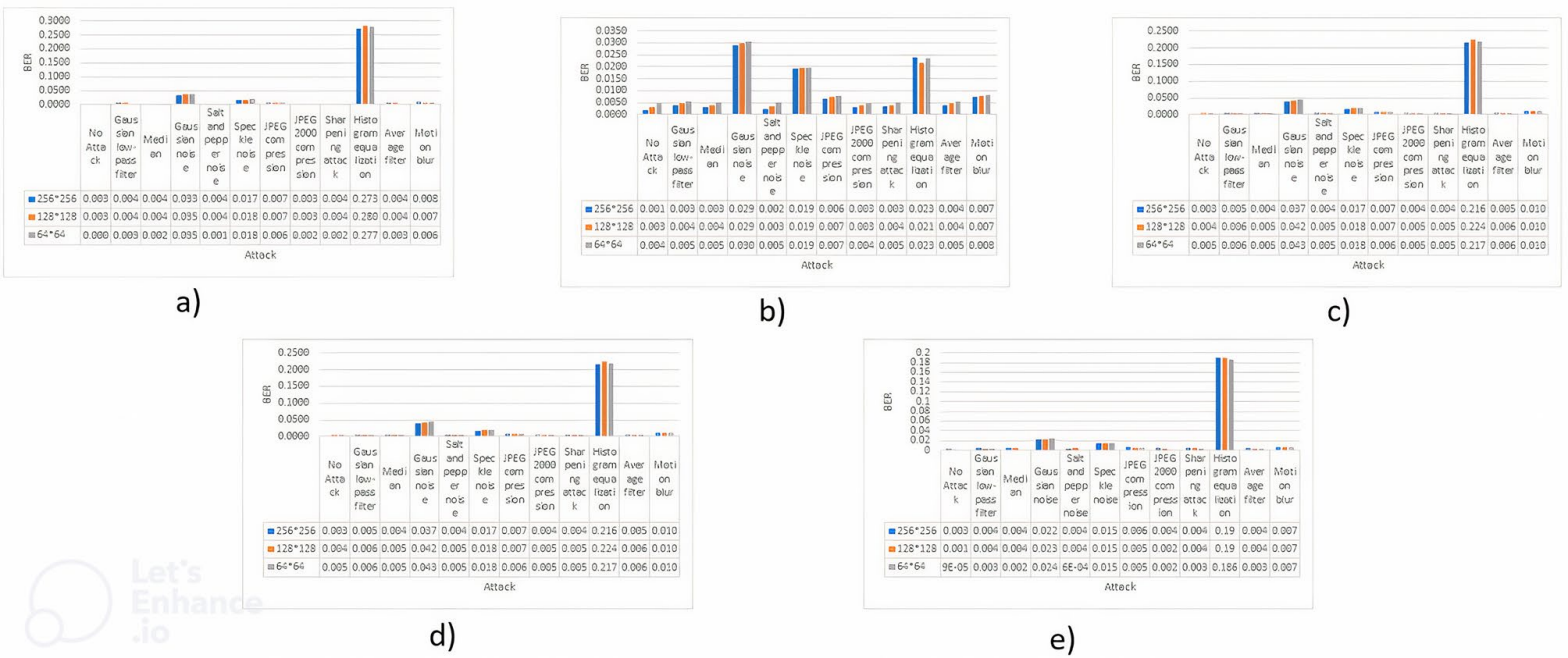
BER values are represented against each attack, values are also shown against all the mentioned attacks and no attack where (**a**) Image 1, (**b**) Image 2, (**c**) Image 3, (**d**) Image 4, (**e**) Image 5.

In this part, our focus shifts towards the analysis of Bit Error Rate (BER) outcomes. The evaluations were performed on the cover photos, as illustrated in Fig. 1, after applying three different watermark sizes described in Fig. 3. The Bit Error Rate (BER), a fundamental measure in the evaluation of watermarking techniques, serves a critical function in evaluating the fidelity of watermarked images in preserving the embedded data relative to the original content. This study thoroughly examines the efficacy of watermarked photos in withstanding a range of potential attacks, as outlined in Table 1. Furthermore, we thoroughly examine their capacity to reduce flaws and guarantee the accurate portrayal of the original content. The results will be concisely displayed in Fig. 8, which will include both numerical data and a graphical depiction of the Bit Error Rate (BER) assessments. This figure will provide a full overview of the watermarking process’s effectiveness in maintaining the accuracy of the preserved information.

After conducting an examination of the results acquired from the five input images, it becomes clear that there is a consistent value close to 0 on the BER value. This effect becomes more pronounced as the dimensions of the watermark become smaller. This phenomenon is present across a wide variety of attacks; nonetheless, it is essential to point out that histogram equalization stands out as a prominent exception to the rule. The general mathematical representation can be written like this:

$$\begin{gathered} \forall {\text{Attacks}} \in \, \{ {\text{Table 3}}\} :{\text{ BER}} \approx 0 \hfill \\ \exists {\text{Attack }}:{\text{ Attack}} = {\text{Histogram equalization s}}.{\text{t}}.{\text{ BER is not close to }}0 \hfill \\ \end{gathered}$$

## Conclusion

Watermarking has long been a tried-and-true method in the realm of data security. In our current digital era, data exchange has witnessed exponential growth year by year across all fields. The medical domain, in particular, is of paramount importance, where various reports are often in image formats. The preservation of the integrity of such data is vital due to its susceptibility to unauthorized alterations and unethical misuse. Over the years, numerous watermarking algorithms have emerged to address this challenge. Evaluating each algorithm’s imperceptibility and robustness is crucial. This paper comprehensively analyses DWT-HD SVD-based watermarking algorithms applied to medical images. Our analysis clearly identifies vulnerabilities that require attention and further refinement. Each algorithm is mathematically examined in detail within its respective section. Notably, the values of NC are consistently close to 1, aligning with expectations, except for Histogram Equalization. The PSNR values increase with decreasing watermark size, with the exception of Histogram Equalization, which yields unfavourable results. SSIM generally reflects good results, barring Gaussian noise, Histogram equalization, and Speckle noise attacks. Additionally, BCR and BER demonstrate strong performance, except for attacks involving Histogram Equalization.

### Future scope

A clear conclusion from the study is that the algorithm could be improved, especially for the attacks that didn’t work very well. One way to make things better is to optimize the algorithm. Adding machine learning and deep learning algorithms together could also help improve the performance of the program.

## Data Availability

The data supporting this study’s findings are available at^27^ Kaggle dataset “COVID-19 Image Dataset” by Pranav Raikokte. The 5 randomly selected images are used in this research paper to generate the results and accessed through the link cited then via data explorer, navigating through the following folder hierarchy: “data explorer” > “Covid19-dataset” > “test” > “Covid”. A Watermark image is generated on paint. Raw data supporting this study’s findings are available from the corresponding author upon request.
